# Ion Mobility–Mass Spectrometry Imaging: Advances in Biomedical Research

**DOI:** 10.3390/biotech14040098

**Published:** 2025-12-18

**Authors:** Mengya Liu, Chi Zhang, Lili Xu, Md. Muedur Rahman, Shoshiro Hirayama, Shuhei Aramaki, Atsushi Baba, Ryo Omagari, Yutaka Takahashi, Tomoaki Kahyo, Mitsutoshi Setou

**Affiliations:** 1 Department of Cellular and Molecular Anatomy, Hamamatsu University School of Medicine, 1-20-1 Handayama, Higashi-Ku, Hamamatsu 431-3192, Shizuoka, Japan; mengyaliu57@gmail.com (M.L.); zhangchi07.pegasus@gmail.com (C.Z.); lilixu2412@gmail.com (L.X.); mrsawpanru@gmail.com (M.M.R.); shirayama@hama-med.ac.jp (S.H.); aramaki@hama-med.ac.jp (S.A.); atbaba@hama-med.ac.jp (A.B.); hotsper0502@gmail.com (R.O.); yutaka.ironman@gmail.com (Y.T.); 2 Preppers Co., Ltd., 1-20-1 Handayama, Chuo-ku, Hamamatsu 431-3192, Shizuoka, Japan; 3 Department of Radiation Oncology, Hamamatsu University School of Medicine, Hamamatsu 431-3192, Shizuoka, Japan; 4 Startup Support and URA Office, Institute of Photonics Medicine, Hamamatsu University School of Medicine, Hamamatsu 431-3192, Shizuoka, Japan; 5 Quantum Imaging Laboratory, Division of Research and Development in Photonics Technology/International Mass Imaging and Spatial Omics Center, Institute of Photonics Medicine, Hamamatsu University School of Medicine, 1-20-1 Handayama, Chuo-ku, Hamamatsu 431-3192, Shizuoka, Japan; 6 International Mass Imaging and Spatial Omics Center, Institute of Photonics Medicine, Hamamatsu University School of Medicine, 1-20-1 Handayama, Chuo-ku, Hamamatsu 431-3192, Shizuoka, Japan

**Keywords:** mass spectrometry imaging (MSI), ion mobility–mass spectrometry (IM-MS), ion mobility–mass spectrometry imaging (IM-MSI), isomerism, biomedical applications

## Abstract

Mass spectrometry imaging (MSI) visualizes the spatial distribution of biomolecules in tissues, whereas ion mobility–mass spectrometry (IM-MS) separates ions through the collision cross-section (CCS) with an inert gas, providing the structural characteristics of isomers. Recent advances have established an integrated workflow, ion mobility–mass spectrometry imaging (IM-MSI), that couples IM with MSI, uniting molecular discrimination with spatial mapping. This synergy has been widely applied in oncology and neuropsychiatric disorders, offering unprecedented insights into biomarker discovery and disease mechanisms. Here, we summarize the principles and classifications of IM-MSI, review their combined biomedical applications, and discuss data processing workflows and commonly used tools.

## 1. Introduction

Mass spectrometry imaging (MSI) is a surface analysis technique capable of simultaneously acquiring molecular and spatial information based on mass-to-charge (*m*/*z*) [1]. With advances in tandem mass spectrometry (MS/MS), MSI distinguishes some isobaric species; differentiating certain isomers requires targeted methods such as multiple reaction monitoring (MRM), often in combination with liquid chromatography or ion mobility. These approaches remain limited: MRM exhibits low throughput. In addition, many MS and MSI systems rely only on *m*/*z* detection and do not include any structural separation step. Ions that share the same mass therefore appear as a single peak and produce overlapping fragment patterns [2]. Therefore, challenges persist in the identification and imaging of isomers.

In biomolecules, isomerism is highly prevalent and plays a significant role in physiological and pathological processes. For instance, amino acids exist as levorotatory and dextrorotatory enantiomers [3]; phospholipids can undergo positional isomerization between *sn-*1 and *sn-*2 sites [4], while fatty acids and cholesterol commonly exhibit double-bond positional isomerism and cis–trans isomerism [5]. Traditionally, methods such as infrared (IR) spectroscopy, ultraviolet (UV) spectroscopy, nuclear magnetic resonance (NMR), and ion mobility–mass spectrometry (IM-MS) have been widely employed to resolve the structures of isomers [6].

When IM-MS is combined with MSI, it overcomes the key limitations of MSI in isomer analysis and complements IM-MS by providing spatial context [7]. The integrated approach, ion mobility–mass spectrometry imaging (IM-MSI), thus emerges as a synergistic strategy with complementary advantages.

This review introduces the principles and applications of MSI and summarizes isomerism with a focus on types common in biomedicine. It then details IM-MSI and recent ion mobility techniques, followed by biomedical applications in oncology and neuropsychiatric disorders. Finally, it provides an overview of data processing and analysis methods for IM-MSI, offering practical guidance for related research.

## 2. Mass Spectrometry Imaging

### 2.1. Definition of Mass Spectrometry Imaging

MSI is an advanced analytical technique that provides the visualization of the spatial distribution of biomolecules, drugs, and metabolites in biological cells and tissues without damaging tissue integrity and thereby causing a loss of spatial information. In this method, analytes are extracted from the sample surface and ionized by various sources (e.g., laser radiation, electrospray, and low-temperature plasma) and detected by the analyzer based on their mass-to-charge (*m*/*z*), along with their coordinate information on the sample surface. A mass spectrum is obtained at each coordinate, and the *m*/*z* values are recorded. Using these *m*/*z* values with their coordinate information, two-dimensional ion density maps, or images, are constructed. MSI has proven to be a valuable tool for investigating the molecular profiling of normal and diseased samples. A key strength of MSI is investigating disease tissue by comparing the histological features obtained from the stained sections using a light microscope with the molecular ion image obtained by MSI, which predicts biomarker discovery and therapeutic target identification [1,8,9]. In the last five years, MSI has advanced with instrumental and methodological development for molecular analysis. Notable instrumental advancements include achieving spatial resolution at 2–5 µm to visualize single cells, a higher resolving power of (>100,000) mass spectrometers to separate adjacent peaks in the mass spectrum, selectivity using selective reactions monitoring (SRM) or MRM mode to identify the target molecules specifically, and integration of post-ionization techniques using laser or plasma to ionize poorly ionized and neutral molecules [10,11,12].

### 2.2. Major MSI Techniques

MSI techniques have been categorized by different criteria like ion sources (i.e., laser and electrospray), tolerance to the surface analytes (i.e., soft and hard ionization), ionization environment (i.e., vacuum and ambient), and using a matrix or being matrix-free. Among these, some well-known MSI techniques have been frequently applied to biomedical science, such as matrix-assisted laser desorption/ionization (MALDI)-MSI (MALDI-MSI), which is a relatively soft ionization technique that can ionize either in a vacuum or an ambient interface system using a laser as an ion source and matrix coating; desorption electrospray/ionization–MSI (DESI-MSI), which is a soft ionization technique that ionizes in an ambient environment using electrospray (i.e., charged solvent) as an ionizing source; secondary ion mass spectrometry (SIMS), which is a hard ionization technique using primary ions to impact on the sample surface, causing desorption of the secondary ions, can provide high spatial resolution, but it is limited by its ionization nature. Many other MSI techniques have been employed using laser irradiation or infrared irradiation and electrospray as ion sources. Of particular interest, for example, are laser ablation electrospray ionization (LAESI), infrared matrix-assisted laser desorption/ionization–MSI (IR-MALDESI-MSI), and laser ablation inductively coupled plasma–MSI (LA-ICP-MSI) [10].

MALDI-MSI: The most widely used technique in MSI, where laser irradiation is used as an ionizing source. A tissue or surface is coated with a chemical matrix that co-crystallizes with the surface analytes and exhibits strong absorption at UV- or IR radiation energy. When the pulsed laser is applied, the matrix helps desorb collectively with the analytes and ionizes the analytes with minimal fragmentation to macromolecules like peptides and proteins (“soft ionization”), but this may cause fragmentation into certain unstable and highly unsaturated compounds [13,14]. The ionization mechanism of MALDI-MSI lies in two proposed models, such as the lucky survivor model, where the analytes and matrix exist in a charged state and are desorbed upon laser irradiation, and the proton transfer model, where a charge transfer reaction between the analytes and matrix happens in the gas phase during ionization [15,16,17,18]. The MALLDI-MSI technique requires multiple steps of sample preparation, which involve cryosection and mounting the tissue section on a special conductive glass slide (i.e., indium tin oxide-coated glass slide), then suitable matrix deposition using the pneumatic spray method or the sublimation method. Different kinds of matrices have been used for MALDI-MSI, for example, 2,5-dihydroxybenzoic acid (DHB) for positive analytes and 9-aminoacridine (9-AA) for negative ion measurement. The advantage of MALDI-MSI lies in its ability to cover a broader range for high-molecular-weight compounds (typically above *m*/*z* 10,000), such as proteins. However, MALDI-MSI is quite unsuitable for low-molecular-weight compound (typically from *m*/*z* 600 to 1000) analysis at the lower mass range (around 50–600 Da), due to the dominance of matrix peaks [19]. MALDI-MSI can be applied for low-molecular-weight compound analysis, but needs prior sample preparation with on-tissue chemical derivatization (OTCD), such as monoamine-containing neurotransmitters and amino acids [20,21]. Alternative MSI techniques, like DESI-MSI, can also be a good choice for low-molecular-weight compound analysis.

DESI-MSI: This is an ambient ionization technique, where a charged solvent (produced after high-voltage application) spray is directed at the sample surface; primary droplets impact the surface, dissolve and extract analytes, and eject secondary droplets that carry (ionized) analytes, which are then directed into the MS inlet [22,23]. This matrix-free technique requires minimal to no sample preparation, allowing DESI to perform analysis within minutes [24,25,26,27]. In contrast, liquid chromatography–mass spectrometry (LC-MS) typically requires 30–120 min of sample preparation plus an additional 10–60 min of chromatographic run time, giving DESI a clear advantage in overall turnaround time [28]. The DESI-MSI technique has been accelerated by substantial instrumental improvement, which includes resolving power, selectively excluding isobaric compounds, the integration of ion mobility to separate isomers in the gas phase, improving sensitivity by instrumental fabrication by the manufacturer, and quantitative mass spectrometry imaging with a rigorous method development [29]. More recently, nanoDESI-MSI gained global interest due to its high spatial resolution [30]. DESI-MSI has now become a powerful tool owing to its operational simplicity, and it is suitable for imaging small molecules such as lipids, neurotransmitters, drugs, and metabolites, which typically fall within an *m*/*z* range of 300–1200 [31]. Rahman et al. applied DESI-MSI in MRM mode to spatial quantitative-MSI (QMSI) of an antimalarial drug, chloroquine, in the kidneys of mice with higher sensitivity and specificity, excluding isomers and isobars, and found that its concentration was highest in the renal pelvis [32]. In addition, Do Huu Chi et al. used DESI-MSI to show that treatment with omega-3 polyunsaturated fatty acids increased nicotinamide adenine dinucleotide levels in the aortic arch of apolipoprotein E-deficient (ApoE^−^/^−^) mice [33].

SIMS: A very sensitive and ultra-high-vacuum hard ionization technique that uses a focused primary ion beam (atomic or cluster) to bombard the sample surface, sputtering off secondary ions for the spatial mapping of analytes. It is inherently surface-sensitive and provides the highest spatial resolution of MSI methods [34]. Time-of-flight SIMS (TOF-SIMS) imaging can achieve a spatial resolution of tens of nanometers (approximately 50–100 nm) or even higher, enabling precise imaging at the subcellular levels to reveal the microscopic distribution of molecules within membranes, cytoplasm, and nuclear regions [35]. For example, a study on plasma membrane heterogeneity used TOF-SIMS to image plasma membrane fatty acids, revealing that they were present and distributed in clusters, and could also be manipulated in clustered forms [36]. Zhang et al. employed a dual-fixation method with glutaraldehyde and uranyl acetate combined with ultrasensitive SIMS imaging to achieve intracellular fatty acid imaging and found that the subcellular distributions were consistent with corresponding organelles [37]. In addition, another study used TOF-SIMS imaging to analyze the conversion of deuterium-labeled α-linolenic acid and linoleic acid within cells, showing that fatty acid treatment significantly increased the levels of their long-chain polyunsaturated fatty acid products and related phospholipids, while also enhancing phospholipid turnover in the plasma membrane [38].

In addition to the above-mentioned MSI techniques, other MSI techniques include laser ablation electrospray ionization–MSI (LAESI-MSI) and infrared matrix-assisted laser desorption–electrospray ionization–MSI (IR-MALDESI-MSI), which are also ambient ionization MSI techniques. The ionization mechanisms rely on the application of a high-energy, mid-IR laser that excites the O-H stretching band of water, which acts as an indigenous IR-MALDI matrix and ablates the analytes. The ablated analytes are then intersected with an electrospray source to generate multiple-charged ions. The sensitivity, throughput, and spatial resolution limit the application of these techniques [39,40]. LESA-MSI is a surface sampling technique considered complementary to high-spatial-resolution imaging technologies. It enables the extraction of analytes from solid substrates by forming liquid micro-bridges between the pipette tip and the sample surface. While proven effective for analyzing intact proteins from diverse sample substrates, it can only provide low-spatial-resolution images (typically 1000 μm) [41].

### 2.3. Current Limitations

Although MSI is widely used to map molecular features in biomedical research, several technical limitations remain. The spatial resolution is restricted by the ionization method, and many platforms cannot reach subcellular detail [42,43]. Throughput is also limited because tissue sections must be scanned pixel by pixel, which increases acquisition time [43]. Sample preparation introduces variability, as sectioning, washing, and matrix applications can alter analyte distribution or ionization efficiency [24,44].

MSI data are further affected by ion suppression and matrix effects, making quantitative analysis difficult [45]. Registration with microscopy or other imaging modalities can be challenging due to tissue deformation during preparation [46]. In addition, most MSI systems probe only the tissue surface, which limits depth information.

Finally, the chemical complexity of tissues creates significant isobaric and isomeric interferences. Many molecules share the same *m*/*z* and therefore appear as a single peak [47]. Without an added separation dimension, MSI alone cannot reliably distinguish these species. Targeted MS/MS strategies such as SRM or MRM can improve specificity but require standards, provide limited coverage, and are not ideal for imaging workflows. These limitations highlight the need for approaches that enhance chemical specificity without sacrificing spatial resolution or throughput [48,49,50,51].

## 3. Isomerism in Biomedicine

Isomers are compounds that share the same molecular formula but differ in structure, and they are widespread in both biological and pharmacological systems [52]. In biomedicine, lipids and amino acids display diverse forms of isomerism, and these subtle structural variations frequently modulate biochemical processes and influence physiological functions [53]. A summary of representative lipid and amino acid isomers, their analytical methods, structural characteristics, and biomedical significance is provided in Table 1.

### 3.1. Isomerism in Biological Molecules

#### 3.1.1. Isomerism in Lipids

Lipids, as essential components of cell membranes, exhibit multiple forms of isomerism, and these structural variations can markedly influence membrane fluidity, signaling pathways, and metabolic regulation [54,55]. Fatty acids often show double-bond positional or cis/trans isomerism; for example, the Δ9 and Δ11 isomers of oleic acid (18:1) display tissue-specific distributions [56], and the cis/trans configuration strongly affects membrane biophysics and metabolic health [57]. Glycerophospholipids also possess *sn-*1/*sn-*2 acyl-chain positional isomers that alter membrane dynamics and cellular signaling [58]. Eicosanoids derived from arachidonic acid contain various positional, functional-group, and tautomeric isomers with distinct roles in inflammation [59,60].

#### 3.1.2. Isomerism in Amino Acids

Amino acids, the building blocks of proteins, primarily exhibit optical isomerism arising from a chiral α-carbon (with glycine as a notable exception) [61]. Proteins are almost exclusively composed of L-amino acids, whereas D-amino acids, though present at low levels in mammals, have important biological functions in specific contexts [62]. D-rine acts as a co-agonist of N-Methyl-D-aspartate (NMDA)-type glutamate receptors and plays a critical role in neurotransmission [63]. D-aspartate accumulates as a marker of protein aging because peptide-bound Asp/Asn residues undergo racemization via succinimide intermediates [64]. The successful identification of D-alanine in human urine demonstrates the significant potential of amino acid enantiomers in screening for chronic kidney disease [65]. Elevated levels of certain D-amino acids are associated with pathological conditions such as chronic kidney disease, cancer, and schizophrenia, highlighting their potential as functional molecules and disease biomarkers [66].

**Table 1:** Representative lipid and amino acid isomers and their biomedical significance.

Category	Biomolecule	Methods	Isomerism Description	Biomedical Significance
Lipids	Oleic Acid (18:1) Δ9 vs. Δ11 [56]	Gas chromatography	Double bond at different positions in the carbon chain	The Δ11 isomer comprises 24–26% in brain, muscle, kidney, and liver tissues, but only 13% in adipose tissue.
Lipids	Oleic Acid (cis vs. trans) [57]	Differential Scanning Calorimetry	Double-bond configuration difference	Trans fatty acids are associated with atherosclerosis and metabolic syndrome. Cis isomers show greater inhibitory effects on insulin secretion and glucose oxidation compared to trans isomers.
Lipids	*sn*-1,2 Positional isomer phospholipids [58]	Isotope labeling + metal ion adduction	Fatty acids are attached to *sn*-1 or *sn*-2 positions of the glycerol backbone	Affects membrane fluidity, signal transduction, and lipid metabolism. Different tissue distributions suggest specific cellular functions.
Amino Acids	L- vs. D-Aspartic acid [62]	LC-MS	Different chirality at the α-carbon	L-form is a natural protein constituent, and D-form is important in bacterial cell walls and neurotransmission. D/L ratios serve as biomarkers for aging and disease.
Amino Acids	L-Serine vs. D-Serine [63]	HPLC	Chiral difference	L-serine participates in metabolism, and D-serine acts as the NMDA receptor co-agonist essential for neurotransmission and synaptic plasticity.
Amino Acids	L- vs. D-Alanine acid [65]	NMR	Chirality difference	Unlike l-amino acids, mostly reabsorbed by kidney tubules, relatively large portions of d-Ala are normally excreted into urine.

### 3.2. Challenges of Isomer Differentiation in Imaging

To investigate molecular structures and distinguish between isomers, a variety of analytical techniques are routinely employed, including UV spectroscopy, IR spectroscopy, NMR spectroscopy, MS, and high-performance liquid chromatography (HPLC) [67]. Each of these methods has inherent limitations: UV spectroscopy lacks structural specificity, IR may fail to resolve fine stereochemical differences, NMR requires relatively large sample amounts and suffers from limited sensitivity, MS may not differentiate isomers with identical fragmentation pathways, and HPLC has a slow separation speed and long analysis time, and it is difficult to separate large molecule isomers [68]. In recent years, IM-MS has emerged as a powerful complementary technique [69]. By separating ions in the gas phase according to differences in collisional cross section (CCS), IM-MS not only enables effective discrimination of stereoisomers but also resolves isobaric and adduct-specific species, reduces spectral congestion, and improves the detectability of low-abundance ions. This orthogonal structural dimension enhances molecular annotation confidence and provides deeper insight into the chemical and biological complexity of isomeric species in situ [70].

## 4. Ion Mobility–Mass Spectrometry

Ion mobility–mass spectrometry (IM-MS) introduces a gas-phase separation step prior to MS analysis. By measuring the mobility of ions under an electric field in an inert gas, IM-MS provides structural information related to the ions’ size, shape, and charge state [69]. Under low-field conditions, ion mobility $K=v_{d}/E$ follows the Mason–Schamp equation, enabling the derivation of the CCS, a parameter highly correlated with three-dimensional molecular structure [71,72].

Ion mobility spectrometry is commonly divided into four types: drift tube ion mobility spectrometry (DTIMS), traveling-wave ion mobility spectrometry (TWIMS), trapped-ion mobility spectrometry (TIMS), and high-field asymmetric waveform ion mobility spectrometry (FAIMS) [73]. Each operates under different electric field regimes and therefore provides distinct capabilities for gas-phase separations.

### 4.1. Drift Tube Ion Mobility Spectrometry (DTIMS)

**Figure 1:**
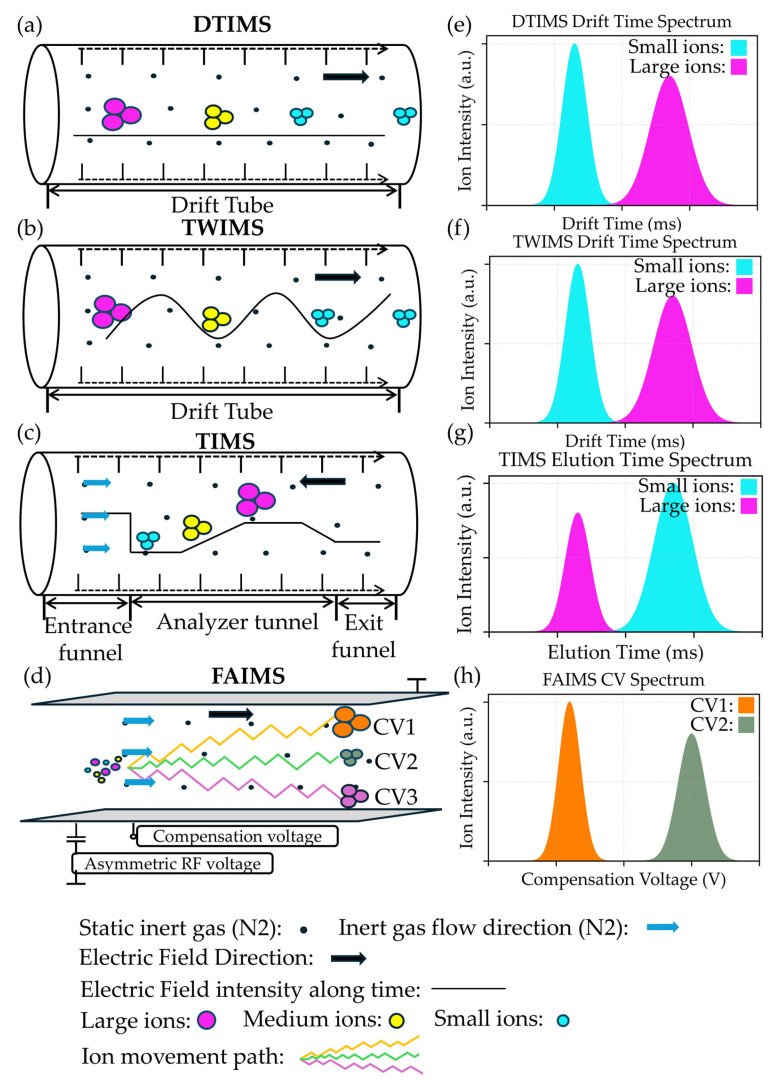
Different types of IM-MS. (**a**–**d**) Mechanisms of IM-MS; (**a**) DTIMS, where the drift tube is filled with static gas, primarily nitrogen. Ions collide with gas molecules and are separated under the influence of an electric field; (**b**) TWIMS, where ions are subjected to a traveling-wave electric field in a gas-filled drift tube, leading to ion separation based on their size, shape, and charge under the influence of the electric field; (**c**) TIMS, where ions are separated under the influence of co-flowing gas and opposing electric fields. (**d**) FAIMS, where ions are separated under the influence of a co-flowing gas and a variable intensity asymmetric waveform electric field. After applying compensation voltage, ions with specific mobility (green dots) are transmitted, while ions with different mobilities (orange and pink dots) are excluded. The black dots in the figure (**a**–**d**) represent buffer gas, typically inert gases (nitrogen). Ions collide with them during drift. (**e**–**h**) Ion drift times in different types of IM-MS. Figure 1e–h are conceptual illustrations of ion drift behavior under typical operating conditions for each IM technology. For clarity, representative constant parameters were assumed (DTIMS: uniform electric field; TWIMS: fixed wave velocity; TIMS: constant ramp rate; FAIMS: fixed DV/CV waveform).
**Panel****Technique****Axis type****Key Feature**(e)DTIMSDrift time (ms)Small ions migrate faster(f)TWIMSDrift time (ms)Small ions migrate faster(g)TIMSElution time (ms)Smaller ions exhibit longer elution times(h)FAIMSCompensation Voltage (V)Different ions have different CVs

DTIMS consists of an ionization region, a pulsed ion shutter, a drift region formed by a drift ring, and a detector [74]. It represents the earliest established form of ion mobility technology. This method injects ions into a tube filled with inert buffer gas via a pulsed ion gate, while applying a uniform electric field across the tube. Ions migrate toward the detector under the influence of the electric field, with their mobility velocity determined by both electric field acceleration and collision resistance from the buffer gas (Figure 1a) [75]. Ions are separated during mobility based on their *m*/*z*, spatial conformation, and interactions with the buffer gas. Compact spherical conformations typically exhibit smaller CCSs and migrate faster, while elongated or extended structures possess larger CCSs and longer mobility times (Figure 1e). In DTIMS under the low-field approximation, the CCS of an ion can be calculated from its mobility using the Mason–Schamp equation [76].

### 4.2. Traveling-Wave Ion Mobility Spectrometry (TWIMS)

TWIMS was proposed by Giles et al. in 2004. Its fundamental structure comprises a stacked ring ion guide (SRIG). Under radial radiofrequency (RF) electric field focusing, ions migrate through a periodic DC pulse potential wave advancing along the axial direction. Ions with compact conformations advance more readily with the wave, while ions with larger surface areas are decelerated by frequent collisions with the buffer gas (Figure 1b). This ultimately generates mobility time differences, enabling the separation of isomers (Figure 1f) [77,78,79]. Unlike DTIMS, TWIMS cannot directly calculate the absolute CCS due to its non-uniform electric field. Calibration typically requires a series of standard compounds with known CCS values, with an accuracy dependent on the rationality of the calibration strategy [77]. TWIMS resolution is primarily influenced by the effective mobility path length [78]. With the introduction of newly designed techniques, such as circular ion mobility (cIM) and Structured Lossless Ion Manipulations, the effective path length has been significantly extended to the meter or even kilometer scale [79].

### 4.3. Trapped-Ion Mobility Spectrometry (TIMS)

TIMS has been a rapidly developing gas-phase ion separation technique in recent years. Its core principle involves ions in the analysis cell being simultaneously pushed toward the outlet by a co-flowing gas and pulled back toward the inlet by a reverse electric field (Figure 1c). These forces reach equilibrium, trapping ions at specific positions (integrated within an ion funnel). As the reverse voltage is gradually reduced, ions with different mobilities are sequentially released, achieving separation based on mobility (Figure 1g). TIMS offers high ion mobility resolution and sensitivity by trapping ions [80,81].

### 4.4. High-Field Asymmetric Waveform Ion Mobility Spectrometry (FAIMS)

FAIMS is a technology fundamentally distinct from traditional IMS platforms in both geometry and separation mechanism. Like TIMS, it relies on a co-flowing gas stream to transport ions through the analysis zone (Figure 1d). Its core mechanism involves distinguishing ions using alternating high-field and low-field voltage waveforms [82,83]. Ions exhibit different mobility rates under high and low electric fields. Only when an appropriate compensation voltage (CV) is superimposed can a specific ion species stably traverse the separation zone and be delivered to the detector; Uncompensated ions deviate from the path and are expelled (Figure 1h). Thus, FAIMS functions more as an electric field-controlled ion filter [84,85]. Unlike DTIMS, TWIMS, or TIMS, FAIMS does not directly provide CCS information, as its separation is not based on absolute mobility measurements. This characteristic limits its application in structural analysis [69].

## 5. IM-MSI Applied in Biomedicine

### 5.1. Establishing the IM-MSI Method in Biomedicine

**Figure 2:**
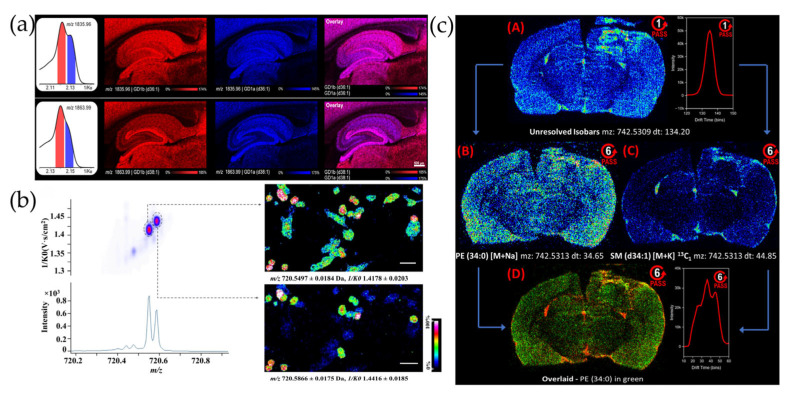
Biomedical applications of IM-MSI. (**a**) MALDI-TIMS-MSI successfully extracted ion mobility profiles for *m*/*z* 1835.96 and *m*/*z* 1863.99 in rat hippocampal tissue and visualized the ion images of the GD1b (red) and GD1a (blue) isomers, as well as their merged image; Adapted with permission from Analytical Chemistry [86]. Copyright© American Chemical Society. License number 6160590081081. (**b**) Ion mobility MSI data collected for isobaric lipids at a narrow mass range of *m*/*z* 720.2 to 720.8 Da (**right** panel) and the spatial distribution of the two isobaric lipids of PE(34:0) ([M+H]^+^, *m*/*z* 720.5497, 1/K0 1.4178) and PC(O-32:0) ([M+H]^+^, *m*/*z* 720.5866, 1/K0 1.4416) based on the combination of their *m*/*z* value and the collisional cross-sectional (CCS) information (**left** panel) in PSC and PANC-1 co-culture samples. Adapted from Nature Communications [88], licensed under CC BY 4.0. (**c**) DESI-cIM-MSI successfully separated the isobaric ions PE(34:0) [M+Na]^+^ and SM(d34:1) [M+K]^+^^13^C_1_ at *m*/*z* 742.53 after six cIM cycles in a rat brain following TBI. Ion images of m/z 742.53 (**A**), PE(34:0) (**B**), SM(d34:1) (**C**), and merge of PE and SM (**D**). Adapted from Analytical Chemistry [31], licensed under CC BY 4.0.

IM-MSI methods were developed to distinguish and visualize isomeric molecules in biomedical samples. MALDI-TIMS-MSI distinguished the isomers of ganglioside (GD1)(d36:) and GD1(d38:1), which are identified as GD1a and GD1b directly in the rat hippocampus according to their different CCSs [86]. GD1a and GD1b showed different hippocampus region-preferential distribution (Figure 2a). GD1a’s and GD1b’s different distributions in the dentate gyrus and subiculum help deepen understanding of the roles of gangliosides in neuronal development and synaptic function. MALDI-TWIMS-MSI distinguished choline, glycerophosphatidylcholine (GPC), and inosine, which have partially overlapping *m*/*z* in a rotenone-treated kidney model [87]. TWIMS-MSI visualized the GPC and choline, especially distributed in the renal medulla, and significantly reduced upon rotenone treatment. That this application revealed a reduction in GPC in the medulla suggests that GPC supplementation could be a new treatment for acute kidney injury.

IM-MSI expands the detectable molecule coverage in biomedical samples. LESA-FAIMS-MSI identified 40 proteins in mouse liver tissue sections compared with only 24 proteins detected with conventional LESA [89]. FAIMS-identified FABP1 is mainly distributed at the outer edge of the liver tissue. This result suggests that the liver may also bind and transport fatty acids from the adjacent peritoneum by FABP1.

### 5.2. IM-MSI Applied in Oncology

IM-MSI helped distinguish and visualize isomeric molecules in cancer samples. MALDI-TIMS-MSI differentiated ions at *m*/*z* 810.5967 into [phosphatidylcholine (PC)(38:4)+H]^+^ and [PC(36:1)+Na]^+^ in adrenocortical carcinoma [90]. MALDI-TIMS-MSI visualized the [PC(38:4)+H]^+^ mainly distributed in the proliferative region of the adrenocortical carcinoma spheroid and the [PC(36:1)+Na]^+^ enriched in the inner necrotic region. IM-MSI revealed that increased fatty acid saturation accelerates adrenocortical carcinoma, suggesting that inhibitors of fatty acid desaturase stearoyl-CoA desaturase 1 may offer therapeutic potential for this malignancy. MALDI-TIMS-MSI was also applied to successfully distinguish phosphatidylethanolamine (PE)(34:0) ([M+H]+, *m*/*z* 720.5497, 1/K0 1.4178) and PC(O-32:0) ([M+H]+, *m*/*z* 720.5866, 1/K0 1.4416) in a co-culture system of human pancreatic cancer (PANC-1) and activated pancreatic stellate cells (PSC) (Figure 2b) [88]. It visualized the PE(34:0) especially distributed in pancreatic cancer.

IM-MSI expands the detectable molecule coverage in cancer tissues and enables clearer identification of lipid species. The MALDI-TIMS-MSI newly identified the ion at *m*/*z* 465.3065 as cholesterol sulfate, which localized at the crypt tops in colorectal cancer (CRC) based on CCS [91]. This helps in understanding the role of the Wnt pathway, which is activated by cholesterol sulfate in CRC pathogenesis. MALDI-TIMS-MSI’s result also suggests that the cholesterol sulfate producer, sulfotransferase 2B1b, could be a treatment target for CRC. MALDI-TIMS-MSI identified 14 cultured breast cancer cell subtypes and 79 lipid subtypes in breast cancer xenograft tissue at the single-cell level [92]. MALDI-TIMS-MSI visualized TG upregulation in (estrogen receptor positive/Progesterone receptor positive/human epidermal growth factor receptor 2 negative) cells, while sphingomyelin (SM) was reduced in triple-negative cells. This result suggests high SM levels and low triacylglycerol (TG) levels as potential new biomarkers for better distinguishing triple-negative breast cancer. DESI and MALDI-TIMS-MSI newly identified and imaged the decrease in N-acetylaspartate (NAA) and increase in adenosine monophosphate (AMP) in the tumor region of a GL261 glioma mouse model [93]. Combined TIMS-MSI suggests a potential role for AMP-activated protein kinase (AMPK) pathway activation in the pathogenesis and progression of glioma. DESI-FAIMS-MSI expanded the number of identified cardiolipin (CL) species from only a few high-abundance forms to a total of 46 species in glioma [94]. DESI-FAIMS-MSI revealed a CL diversity reduction in astrocytoma (AST) and glioblastoma (GBM) compared to the normal cortex. Furthermore, IM-MSI visualized that long-chain CL species were nearly absent in GBM, while short-chain CL species were enriched. DESI-FAIMS-MSI’s result indicates that CL metabolism reprogramming heavily affects the GBM pathogenesis and suggests the enzymes involved in cardiolipin metabolism could be a potential therapeutic target.

### 5.3. IM-MSI Applied in Neuropsychiatric Disorders

IM-MSI improved the distinguishing and visualization of isomeric molecules in neuropsychiatric disorders. MALDI-TIMS-MSI separated isobaric lipids as phosphatidic acid (PA), phosphatidylserine (PS), and PE in Alzheimer’s Disease model mice [95]. MALDI-TIMS-MSI revealed PA(16:0/16:0) and PE(18:1/18:0) colocalized with the plaque core. IM-MSI enables a deeper understanding of how lipid species with distinct biological activities contribute to the pathological polymorphism of AD, thereby advancing our elucidation of its underlying mechanisms. DESI-cIM-MSI successfully separated the isobaric ions PE(34:0) [M+Na]^+^ and SM(d34:1) [M+K]^+^^13^C_1_ at *m*/*z* 742.53 after six cIM cycles in a rat brain following traumatic brain injury (TBI). The spatial maps further revealed that PE(34:0) was distributed across the entire gray matter, whereas SM(d34:1) was concentrated in the hippocampus, hypothalamus, and around the lesion area (Figure 2c) [31]. DESI-cIM-MSI indicates the myelin repair processes occurring along functionally interconnected neural circuits after TBI. MALDI-TWIMS-MSI identified and imaged isobaric lipids such as lysophosphatidylethanolamine (LPE)(22:6) and lysophosphatidylserine (LPS)(18:0) [M−H]^−^ ions, as well as PC(34:1) [M+Cl]^−^ and PE(40:4) [M−H]^−^ ions, by using CCS values in a Smith–Lemli–Opitz syndrome (SLOS) mouse model [96]. MALDI-TWIMS-MSI showed different lipid distribution patterns in SLOS, yet the spatial resolution was not enough to identify the detail in the brain region. MALDI-TIMS-MSI separated PC and SM with close *m*/*z* by TIMS in multiple sclerosis research. The study revealed a PC and SM reduction in lesion regions compared to adjacent normal white matter in multiple sclerosis research [97]. This helped understand the membrane’s biophysical properties and SM-induced apoptosis function in MS pathogenesis and progress. Taken together, these studies demonstrate the expanding utility of IM-MSI in revealing isomer-specific molecular distributions and disease-related biochemical alterations. A structured summary of these applications is presented in Table 2.

### 5.4. Advantages and Limitations of IM-MSI

Across oncology and neuropsychiatric applications, IM-MSI provides three key advantages: an increased number of distinguishable ion features enabled by the gas-phase separation of isobaric and isomeric species, access to CCS values that add a structural dimension to MSI data, and while retaining the advantages above, it additionally provides the inherent spatial information at tissue, cellular, and even subcellular scales, thereby further enhancing chemical specificity and confidence in molecular assignments.

However, several limitations must also be acknowledged. First, improving ion mobility resolution or extending ion paths often comes at the cost of longer acquisition times and increased ion losses, which can reduce sensitivity for very-low-abundance analytes in untargeted IM-MSI experiments [98,99]. Second, the quantitative comparability of CCS values across instruments still requires careful calibration and standardized workflows, particularly for TWIMS and TIMS devices [100,101]. Third, the multidimensional nature of IM-MSI data (*m*/*z*, drift time/CCS, intensity, and x-y-z position) demands advanced data processing, robust peak picking, and machine learning-based classification, which are not yet fully standardized [12,43]. Finally, there is an inherent trade-off between spatial resolution, mass resolution, and ion statistics, especially in single-cell or subcellular imaging regimes [102,103].

Recognizing these strengths and limitations is crucial for the rational design of IM-MSI studies. As ion transmission optics, high-efficiency ion mobility devices, and computational tools continue to improve, it is expected that IM-MSI will become an increasingly powerful platform for spatial omics in both basic research and clinical translation.

## 6. Analytical Tools for Ion Mobility Spectrometry–Mass Spectrometry

IM-MSI data processing requires software that can simultaneously manage spatial coordinates (x-y), *m*/*z* values, ion-mobility information (drift time or CCS), and MS/MS spectra. In contrast to IM-MS workflows, which operate on chromatographic or spectral data, IM-MSI tools must treat ion mobility as an additional image-forming dimension. This imposes distinct computational challenges, including substantially larger multidimensional datasets, higher memory requirements, and the need for algorithms capable of reconstructing mobility-resolved ion images.

Developed tools currently used for IM-MSI data processing, including SCiLS Lab and HDI. SCiLS Lab (Bruker, Bremen, Germany), which provide drift-time-resolved ion images, 4D peak picking, segmentation, and statistical analysis for timsTOF fleX datasets [104]. Waters High-Definition Imaging (HDI) Software (version 1.4; Waters Corporation, Milford, MA, USA) supports DESI HDMS MSI data processing with CCS-based annotation [105]. Both platforms remain vendor-specific and closed-source, limiting cross-instrument interoperability and algorithmic customization.

Recently, the under-development MSI software Cardinal (version 3) has supported the import, preprocessing, segmentation, and statistical analysis of large-scale imaging [106]. However, it lacks IM-specific functionality and does not support drift time/CCS feature extraction. It can only process imzML data containing IM information as standard MSI files. Additionally, TIMSCONVERT (Bruker, Bremen, Germany) was developed to convert TIMS data to imzML [107]. The open-source community has started exploring IM-MSI workflows based on toolchains such as TIMSCONVERT + Cardinal (e.g., Open Source Spatial Reactomics, OSSpRe), to process and perform downstream analysis on TIMS-coupled MSI data [108].

Current IM-MSI workflows remain fragmented. A major limitation is the absence of an integrated processing pipeline. Tools such as MZmine 3 (version 4.3) and OpenMS (version 3.5) were developed for IM-MS and include IM-aware feature detection. However, their imaging modules remain aligned with traditional MSI paradigms and currently do not expose a full IM-MSI pipeline.

Collectively, while some developed platforms provide primary solutions for current workflows, the IM-MSI field still lacks comprehensive and algorithm-rich analysis pipelines. These gaps highlight an important opportunity for future tool development, particularly in areas of standardized data formats, multi-dimensional feature extraction, CCS-informed annotation, and integrated computational frameworks tailored to IM-MSI.

## 7. Prospects

Given the widespread occurrence of isomerism in biomedicine, we hold high expectations for the future development of IM-MSI in medical research and clinical applications.

IM-MSI demonstrates exceptional applicability in lipid research. Lipids are highly heterogeneous biomolecules, where positional, cis–trans, and enantiomeric isomerism play crucial roles in membrane structure, signal transduction, and metabolic regulation [109]. Recent studies increasingly highlight the critical role of lipids and their metabolites in tumor microenvironment reprogramming and neurodegenerative diseases [110,111]. These molecules frequently function as signaling molecules involved in disease onset and progression. We anticipate that IM-MSI will reveal the spatial distribution and dynamic metabolic characteristics of these signaling molecules at the tissue and cellular levels. This will aid in deciphering the pathological mechanisms of complex diseases and open new avenues for the diagnosis and treatment of inflammation-related disorders.

In neurodegenerative diseases, the conformational diversity of pathology-associated proteins is closely linked to disease type, pathogenesis, and progression rate [112]. For instance, Jeacock et al. have reported the use of CCS values to classify and identify conformational differences in N-acetylated α-synuclein (α-syn) [113]. Thus, IM-MSI holds potential for exploring the structural heterogeneity of pathological proteins and their spatial distribution characteristics within brain tissue. We anticipate that future IM-MSI technology will systematically reveal the conformational lineages of abnormal proteins in diseases such as Alzheimer’s disease, Parkinson’s disease, and multiple system atrophy, establishing connections between their structural characteristics and disease progression. This will not only deepen our understanding of the onset and development of neurodegenerative diseases but also provide new references for formulating precision intervention strategies.

Furthermore, in the study of traditional Chinese medicine herb ingredients, IM-MSI holds great potential. IM-MSI enables the discrimination of structural isomers and isobars, as well as the visualization of bioactive components and metabolites within herbal tissues. These capabilities provide novel insights into the synergistic mechanisms, pharmacodynamic material basis, and metabolic transformations of multi-component TCM systems. In the future, the combination of high-throughput TIMS/PASEF technologies, comprehensive CCS databases, and intelligent data-processing algorithms is expected to further enhance the application of IM-MSI in quality control, bioactive compound screening, authenticity verification, and in vivo metabolism studies, offering a powerful tool to accelerate the modernization and precision research of traditional Chinese medicine herbs [114,115].

The integration of artificial intelligence and trapped-ion mobility spectrometry (AI-TIMS) provides practical solutions to current IM-MSI challenges, including mobility-aware peak picking, denoising, CCS prediction, and isobar annotation. Yet supervised models depend heavily on training-set chemistry; drug molecules often fall outside the structural space of endogenous lipids and metabolites. Developing chemically diverse reference datasets and domain-adaptation strategies will therefore be essential for reliable AI-assisted IM-MSI interpretation.

As IM-MSI begins to reveal spatially distinct distributions of isomeric lipids and metabolites, a major future challenge is the integration of these structural maps with protein or transcript expression patterns. Instead of relying on serial sections for CosMx, Xenium, or other spatial transcriptomics platforms, future workflows will require technologies capable of acquiring multiple spatial omics layers from the same tissue section, as well as computational frameworks that can align high-resolution transcriptomic data with IM-MSI ion images. Together, these developments outline realistic and technically grounded directions for advancing IM-MSI beyond its current limitations.

## Figures and Tables

**Table 2:** IM-MSI applied in biomedicine.

Disease/Model/ Application	Ionization (Imaging Unit)	Ion Mobility Spectrometry	Observed Molecules/Isomers	Main Findings
IM-MSI method establishment				
Gangliosides (Rat hippocampus & spinal cord) [86]	MALDI	TIMS	GD1a/b(d36:1)*:m*/*z* 1835.96 GD1a/b(d38:1):*m*/*z* 1863.99	Distinct a- vs. b-isomer distributions across hippocampal regions
Drug metabolism/toxicity (rotenone in the kidney) [87]	MALDI	TWIMS	Choline: *m*/*z* 104.1071 [M+H]^+^ GPC: *m*/*z* 296.0665 [M+K]^+^) Inosine: *m*/*z* 307.0445 [M+K]^+^	Distinguished choline, GPC, and inosine in the kidney
Protein imaging (mouse liver) [89]	LESA	FAIMS	40 proteins	Specific protein FABP1 observed through FAIMS
IM-MSI applied in Oncology				
H295R(MCTS) [90]	MALDI-2	TIMS	[PC(38:4)+H]^+^ vs. [PC (36:1)+Na]^+^: *m*/*z* 810.5967	Enhanced lipid metabolite signaling and distinguishing isomers
PNAC-1/PSC [88]	MALDI	TIMS	PE(34:0) ([M+H]^+^, *m*/*z* 720.5497); PC (O-32:0) ([M*+*H]^+^, *m*/*z* 720.5866)	Revealing metabolic heterogeneity among cells.
Colorectal cancer (CRC) [91]	MALDI	TIMS	Cholesterol sulfate increased and glutathione decreased	Cholesterol sulfate plays a crucial role in tumor progression
Breast cancer [92]	MALDI/MALDI2	TIMS	Lipids	Identified 79 different lipids present in different ratios in all 14 cultured breast cancer cell subtypes
Glioblastoma (GL261 mouse) [93]	DESI/MALDI	TIMS	NAA, AMP, fatty acids	Tumor: NAA decreased, AMP increased, de novo FA synthesis/elongation increased
Glioma (AST, GBM) [94]	DESI	FAIMS	Cardiolipin (CL)	CL diversity reduced; long-chain CL(78:12) absent in GBM; AST showed higher Δ9 isomer ratios, CL as a potential biomarker and therapeutic target
IM-MSI applied in neuropsychiatric disorders				
Alzheimer’s disease (AD, tgAPPswe) [95]	MALDI	TIMS	PA(16:0/16:0): *m*/*z* 647.464 PS(18:0/22:6): *m*/*z* 834.525 PE(22:6/22:6): *m*/*z* 834.51	Plaque lipids altered; TIMS localized PA, PS, PE
Traumatic brain injury (TBI, rat) [31]	DESI	cIM	PE(34:0)+Na vs. SM (d34:1) +K: *m*/*z* 742.531	Revealed lipid remodeling and injury-specific localization
Smith–Lemli–Opitz syndrome (SLOS, Dhcr7-KO) [96]	MALDI	TWIMS	LPE(22:6) [M−H]^−^ vs. LPS (18:0) [M−H]^−^: *m*/*z* 524.279 vs. *m*/*z* 524.302, PC(34:1) [M+Cl]^−^ vs. PE (40:4) [M−H]^−^: *m*/*z* 794.547 vs. *m*/*z* 794.564	Sterol precursor accumulation in Dhcr7-KO mouse brain; CCS resolved isotopologues
Multiple sclerosis (MS) [97]	MALDI	TIMS	PC, SM	PC and SM reduced in lesions; *m*/*z* 714.602 isomers resolved by CCS

## Data Availability

No new data were created or analyzed in this study.

## Abbreviations

The following abbreviations are used in this manuscript:

AD	Alzheimer’s Disease
AMP	Adenosine Monophosphate
AST	Astrocytoma
cIM	Circular Ion Mobility
CCS	Collision Cross-Section
CL	Cardiolipin
CRC	Colorectal Cancer
CV	Compensation Voltage
DESI-MSI	Description Electrospray/Ionization–Mass Spectrometry Imaging
DTIMS	Drift Tube Ion Mobility Spectrometry
DHB	2,5-dihydroxybenzoic acid
FAIMS	High-Field Asymmetric Ion Mobility Spectrometry
GBM	Glioblastoma
GD1	Ganglioside D1
GPC	Glycerophosphocholine
HPLC	High-Performance Liquid Chromatography
HDI	High-Definition Imaging
IM	Ion Mobility
IM-MSI	Ion Mobility–Mass Spectrometry Imaging
IM-MS	Ion Mobility–Mass Spectrometry
IMS-MSI	Ion Mobility Spectrometry–Mass Spectrometry Imaging
IR	Infrared Spectroscopy
IR-MALDESI-MSI	Infrared Matrix-Assisted Laser Desorption Electrospray Ionization–Mass Spectrometry Imaging
IR-MALDI	Infrared Matrix-Assisted Laser Desorption/Ionization
KO	Knockout
LC-MS	Liquid Chromatography–Mass Spectrometry
LA-ICP-MSI	Laser Ablation Inductively Coupled Plasma–Mass Spectrometry Imaging
LAESI	Laser Ablation Electrospray Ionization
LAESI-MSI	Laser Ablation Electrospray Ionization–Mass Spectrometry Imaging
LESA-FAIMS	Liquid Extraction Surface Analysis–High-Field Asymmetric Ion Mobility Spectrometry
LESA-MSI	Liquid Extraction Surface Analysis–Mass Spectrometry Imaging
LPE	Lysophosphatidylethanolamine
LPS	Lysophosphatidylserine
MSI	Mass Spectrometry Imaging
MS/MS	Tandem Mass Spectrometry
MALDI-MSI	Matrix-Assisted Laser Desorption/Ionization–MSI (mass spectrometry imaging)
MALDI-MSI-IMS	Matrix-Assisted Laser Desorption/Ionization–Mass Spectrometry Imaging–Ion Mobility Spectrometry
MCTS	Multicellular Tumor Spheroid
MRM	Multiple Reaction Monitoring
NAA	N-Acetylaspartate
NanoDESI-IM-MS	Nanospray Desorption Electrospray Ionization–Ion Mobility–Mass Spectrometry
NMDA	N-Methyl-D-Aspartate
NMR	Nuclear Magnetic Resonance
OTCD	On-tissue Chemical Derivatization
PA	Phosphatidic Acid
PC	Phosphatidylcholine
PE	Phosphatidylethanolamine
PS	phosphatidylserine
PANC-1	Human Pancreatic Cancer
PSC	Pancreatic Stellate Cells
QMSI	Quantitative-MSI
RF	Radial Radiofrequency
SIMS	Secondary Ion Mass Spectrometry
SLOS	Smith–Lemli–Opitz Syndrome
SM	Sphingomyelin
SRIG	Stacked Ring Ion Guide
SRM	Selected Reaction Monitoring
TBI	Traumatic brain injury
TG	Triacylglycerol
TIMS	Trapped-Ion Mobility Spectrometry
TOF-SIMS	Time-of-Flight Secondary Ion Mass Spectrometry
TWIMS	Traveling-Wave Ion Mobility Spectrometry
UV	Ultraviolet Spectroscopy
